# Risser growth plate injury in unstable paediatric pelvic fractures: a multicentre retrospective study

**DOI:** 10.1186/s10195-026-00935-5

**Published:** 2026-05-30

**Authors:** Michel Oransky, Angelo Gabriele Aulisa, Andrés Roncoroni, Adrien Roa Zoppi, Mariangela Mata, Mohamed Abdelwahed Rohayem, Francesco Falciglia, Renato Maria Toniolo

**Affiliations:** 1 Private Practitioner, Rome, Italy; 2 U.O.C. of Orthopaedics and Traumatology, Bambino Gesù Children’s Hospital, IRCCS, P.zza S. Onofrio 4, 00165 Rome, Italy; 3 Department of Human Sciences, Society and Health, University of Cassino and Southern Lazio, 03043 Cassino, FR Italy; 4 Hospital Nacional de Pediatría Juan P. Garrahan, Buenos Aires, Argentina; 5 Unidad de Cirugia Reconstructiva de Cadera Pelvis y Acetábulo, Policlinica Metropolitana, Caracas, Venezuela; 6 Orthopedic Surgery Department, Tanta University Hospitals, Tanta, Egypt

**Keywords:** Paediatric pelvic fracture, Risser growth plate injury, Physeal trauma, Paediatric pelvis

## Abstract

**Introduction:**

Unstable pelvic fractures in children are rare but severe injuries and are associated with high-energy trauma. Unlike adults, children have open growth plates, particularly the Risser growth plate (RGP), which is vulnerable to injury. The presence of growth plates, such as the triradiate cartilage and Risser’s plate, represents point of vulnerability that determines distinctive fracture patterns, similar to those seen with Salter–Harris growth plate injuries.

Lateral compression fracture is the most common mechanism and results in an irreducible crushing of the sacral alae, causing a rotational deformity. Both vertical and lateral compression displaced pelvic fractures require careful evaluation of the posterior iliac apophysis-associated injury.

Failure to treat Risser growth plate injury (RGPI) can lead to severe long-term problems such as limb length discrepancy and scoliosis. This study aimed to determine how commonly RGPI occurs in unstable paediatric pelvic fractures, link it to specific fracture patterns and propose a subgroup type to improve treatment.

**Materials and methods:**

This multicentre retrospective study included 40 children aged up to 12 years with unstable pelvic fractures (AO/OTA 61B and 61C). The patients were treated between 1987 and 2022 at trauma centres in Italy, Argentina and Venezuela. We reviewed patient demographics, injury mechanisms and computed tomography (CT) scans to classify fractures using the AO/OTA system and identify RGPI. Statistical analysis was used to explore links between RGPI and specific fracture features.

**Results:**

Forty children (25 males; mean age 7.3 ± 3.6 years) were included. RGPI occurred in 26/40 patients (65%), with 19/21 cases (90.4%) in AO/OTA type C fractures. RGPI significantly correlated with complex posterior fracture-dislocations (*p* = 0.001) and AO/OTA type C injuries (*p* = 0.0007). Three RGPI types were identified: type 1 (minimally displaced), type 2 (avulsed RGP with sacroiliac [SI] joint disruption) and type 3 (bilateral lesions). In total, 11 of 13 patients (84.6%) with at least 2 years of follow-up developed deformities.

**Discussion and conclusions:**

RGPI is a common and critical part of unstable paediatric pelvic fractures, especially severe ones. In patients below the age of 7 years old with displaced and unstable pelvic fractures, the Risser’s growth plates and posterior iliac apophysis are involved in 50% of cases. The Tile/AO classification, adapted to include concurrent injuries of the growth plates, offers a useful framework for treatment planning. Although the Torode and Zeig classification remains the standard, it is incomplete because it does not consider injuries related to the Risser growth plate. Our findings suggest that unrecognised or inadequately reduced RGPI causes severe, progressive functional deformities, such as limb length discrepancy, rather than mechanical instability. Although further prospective validation is needed, we hypothesize that anatomical reduction of these specific physeal injuries should be considered to prevent severe growth arrest. Our proposed classification seeks to improve upon traditional frameworks by incorporating this critical structure.

**Level of Evidence:**

Level 4 (case series).

## Introduction

Unstable pelvic fractures in children are rare but can be potentially devastating injuries, usually caused by high-energy trauma such as motor vehicle accidents, pedestrian collisions or falls from height [1–4]. Unlike the adult pelvis, the immature pelvis, characterised by open growth plates, has unique biomechanical properties. Its greater elasticity and the significant strength of its ligaments mean that when subjected to traumatic forces, the failure point is often not the bone or the ligament itself, but rather the cartilaginous structures. The growth plates (physes), especially the triradiate cartilage of the acetabulum and the iliac apophysis, are the pelvis’s ‘locus minoris resistentiae’, resulting in distinctive modes of failure [5]. Among these physeal disruptions, avulsion of the iliac apophysis and the associated Risser growth plate injury (RGPI) were first described by Oransky and Kenawey [6, 7] as a critical and unique aspect of paediatric pelvic fractures. Failure to recognise and properly treat an RGPI can lead to severe long-term consequences, including growth arrest of the hemipelvis, which may result in limb-length discrepancy, secondary scoliosis and considerable disability [8–10]. Despite the potential seriousness of these sequelae, RGPI remains an often underestimated or unrecognised part of paediatric pelvic trauma. Its true incidence has never been precisely established, and its relationship with various patterns of pelvic instability (rotational or vertical) remains unclear [11, 12].

Currently, paediatric pelvic ring injuries are primarily classified using the Torode–Zeig system [13]. Although widely adopted, this classification is fundamentally incomplete for the immature skeleton because it completely ignores the Risser growth plate (RGP) nucleus. This omission creates a significant conceptual gap. Epiphyseal separations in children require specific attention: unlike adult pelvic trauma, where the main surgical aim is to restore mechanical stability, inadequate reduction of paediatric physeal injuries can directly lead to severe growth disturbances and functional deformities.

The purpose of this retrospective multicentre study was to determine the prevalence of associated RGPI in a series of displaced rotational and vertically unstable paediatric pelvic fractures and to propose subgroup types on the basis of location, displacement and bilaterality. It helped in recognising and treating the condition, surpassing and integrating supplementary methods with traditional systems, depending on the RGP injury. Furthermore, we report two cases of a bending deformity of the iliac wing associated with the cranial portion of the hemipelvis, a mode of failure not previously described.

## Materials and methods

This retrospective study was carried out at three academic Level 1 trauma centres in Italy, Argentina and Venezuela. All consecutive patients treated for displaced pelvic fractures between 1987 and 2022, aged up to 12 years, with an immature pelvis confirmed by the presence of open triradiate cartilage and with unstable pelvic fractures (AO/OTA 61B and 61C) were eligible for inclusion.

The diagnostic protocol for pelvic fractures at all centres included plain radiographs and a computed tomography (CT) scan. The primary diagnosis was often clear from radiographs; CT scans were essential for detailed characterisation of injuries, including the identification of RGPI, thus ensuring diagnostic consistency throughout the study period.

While magnetic resonance imaging is highly sensitive for physeal injuries, it is largely impractical and contraindicated in the acute management of unstable, high-energy paediatric pelvic ring traumas, where rapid, standardised multiplanar CT is the gold standard for life-saving decision-making and surgical planning.

Patients with pathological fractures, endocrine disturbances, children over 12 years old and non-displaced pelvic fractures were excluded. Demographic data, including age, sex and mechanism of injury, were collected retrospectively from the medical records. The radiographic assessment, including the AO/OTA classification and the proposed RGPI subclassification, was performed by a highly specialised multidisciplinary team. All authors are expert paediatric orthopaedic surgeons, and the assessment team included three senior, highly experienced pelvic trauma surgeons. These experts reached consensus to assign radiographic classifications, ensuring clinical accuracy and consistency.

Discrete lesions were documented and categorised into those affecting the anterior and posterior pelvic ring. Posterior lesions were recorded as sacroiliac joint (SIJ) dislocation, fractures through the sacrum, transiliac fracture dislocation of the SIJ, trans sacral fracture dislocation of the SIJ and ilium fractures. Anterior injuries encompassed symphysial diastasis, unilateral pubic rami fracture, bilateral pubic rami fractures, or a combination of pubic rami and symphysial injuries. Avulsion of the iliac apophysis and RGPI, when present, was identified on CT scans. At the final follow-up, weight-bearing panoramic radiographs were reviewed to evaluate deformity, pelvic obliquity and compensatory scoliosis.

### Statistical analysis

Descriptive statistics were used to summarise patient demographics (age, sex) and fracture characteristics (mechanism of injury, AO/OTA pelvic fracture classification, anterior lesion, posterior lesion and RGPI). Continuous data were tested for normality using the Shapiro–Wilk test. Normally distributed variables were reported as means with standard deviations and compared using Student’s *t*-test. Non-normally distributed variables were reported as medians with interquartile ranges (IQR) and compared using the Mann–Whitney *U* test. Categorical variables were expressed as frequencies and percentages and compared using the Chi-squared or Fisher’s exact test. Statistical significance was defined as a *p*-value < 0.05. All analyses were performed using Python 3 (Wilmington, DE, USA).

## Results

The study included 40 paediatric patients: 25 males (62.5%) and 15 females (37.5%). The mean age at injury was 7.3 years (range: 1–12; SD: 3.6). Injury mechanisms consisted of vehicle rollover in 22 cases (55%), motor vehicle collision in 11 cases (27.5%) and fall from height in 7 cases (17.5%). Associated skeletal fractures were found in 13 patients (32.5%). Using the AO/OTA classification system, pelvic injuries were categorised as follows: type B injuries included B1.1 (*n* = 3), B2.1 (*n* = 6), B2.2 (*n* = 6), B2.3 (*n* = 2), B3.1 (*n* = 1) and B3.3 (*n* = 1); and type C injuries included C1.2 (*n* = 9), C2.1 (*n* = 1), C2.2 (*n* = 2), C3.1 (*n* = 6), C3.2 (*n* = 2) and C3.3 (*n* = 1).

Posterior lesions included pure sacroiliac (SI) joint dislocations in 21 cases (6 bilateral), SI dislocations with contralateral windswept-type lesions in four cases, crescent-type fracture-dislocations in eight cases and sacral ala compression fractures involving growth plate nuclei in eight cases. Risser growth plate injury (RGPI) was found in 26 patients (65%), with two cases being bilateral. No significant links were observed between RGPI and age, sex or age group (≤7 versus >7 years) (Table 1). C-type pelvic fractures were significantly more common in patients with RGPIs (73.1%, *n* = 19) compared with patients with non-RGPIs (14.3%, *n* = 2). RGPI showed strong associations with C-type AO/OTA fractures (*p* = 0.0007) and complex posterior fracture-dislocations (*p* = 0.001) (Table 2).

**Table 1:** Demographic and clinical characteristics by Risser growth plate injury (RGPI)

	No RGPI (*n* = 14)	RGPI (*n* = 26)	*p*-value	Test
Age (years)				
Mean ± SD	8.4 ± 2.6	7.3 ± 3.5	0.205	Mann–Whitney *U*
Median (range)	9 (3–12)	7 (1–12)		
Sex				
Male, *n* (%)	9 (64.3%)	16 (61.5%)	0.860	Chi-square
Female, *n* (%)	5 (35.7%)	10 (38.5%)		
Age group				
>7 years, *n* (%)	10 (71.4%)	11 (42.3%)	0.154	Chi-square
≤7 years, *n* (%)	4 (28.6%)	15 (57.7%)

**Table 2:** Injury patterns by Risser growth plate injury (RGPI)

	No RGPI (*n* = 14)	RGPI (*n* = 26)	*p*-value	Test
AO/OTA classification			**0.0007**	Fisher’s exact
B-type, *n* (%)	12 (85.7%)	7 (26.9%)		
C-type, *n* (%)	2 (14.3%)	19 (73.1%)		
Anterior lesion			0.2710	Fisher’s exact
Bilateral rami, *n* (%)	4 (28.6%)	3 (11.5%)		
Synphysis diastasis, *n* (%)	2 (14.3%)	11 (42.3%)		
Unilateral rami, *n* (%)	5 (35.7%)	5 (19.2%)		
Other, *n* (%)	1 (7.14%)	4 (15.4%)		
No lesion, *n* (%)	2 (14.3%)	3 (11.5%)		
Posterior lesion			**0.001**	Fisher’s exact
Sacral fracture, *n* (%)	10 (71.4%)	1 (3.85%)		
SI dislocation, *n* (%)	2 (14.3%)	9 (34.6%)		
Bilateral SI dislocation, *n* (%)	1 (7.14%)	6 (23.1%)		
Fracture-dislocation, *n* (%)	0 (0%)	10 (38.5%)		
No lesion, *n* (%)	1 (7.14)	0 (0%)		

Bold values indicate statistically significant differences (p 0.05)

### Risser growth plate injuries (RGPI)

The literature on paediatric pelvic injury classification remains limited, with no existing system that accounts for RGPIs [14, 15].

**Fig. 1:**
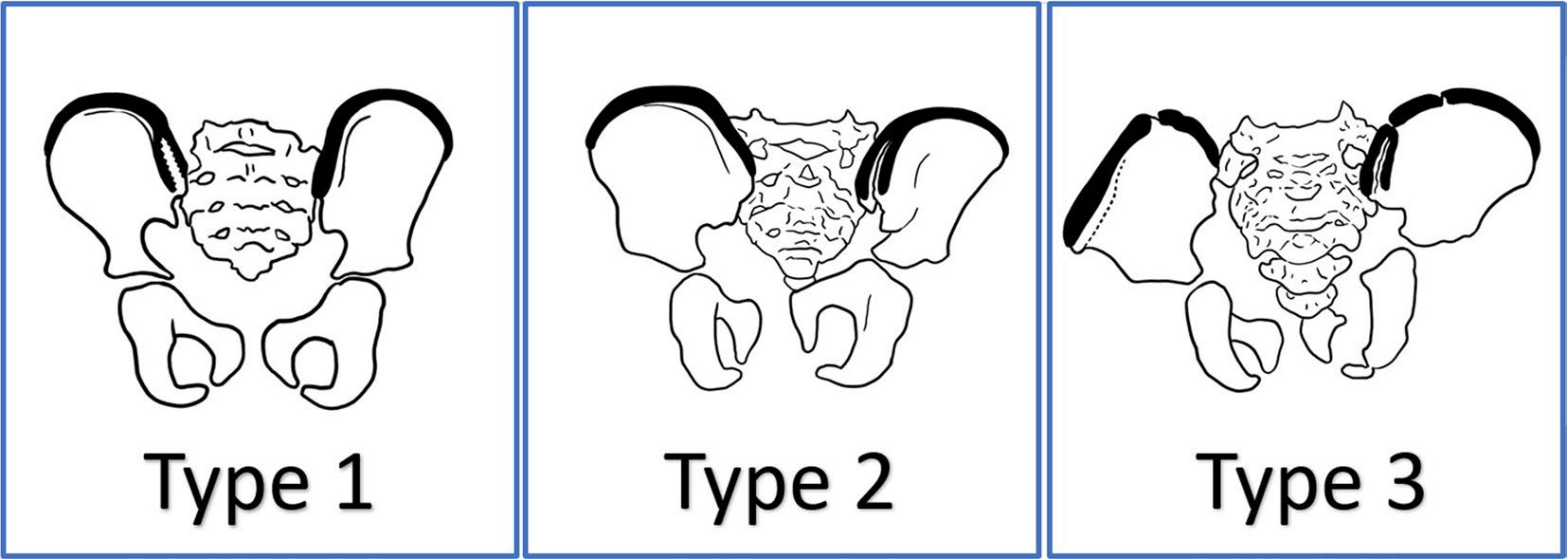
Visual representation of the classification patterns (types 1–3), designed to supplement radiographic findings and enhance diagnostic clarity

On the basis of our 40 cases of unstable paediatric pelvic fractures, we identified and proposed three subgroups for Risser growth plate injuries (RGPI) on the basis of displacement features and related pathoanatomy (Fig. 1).

**Fig. 2:**
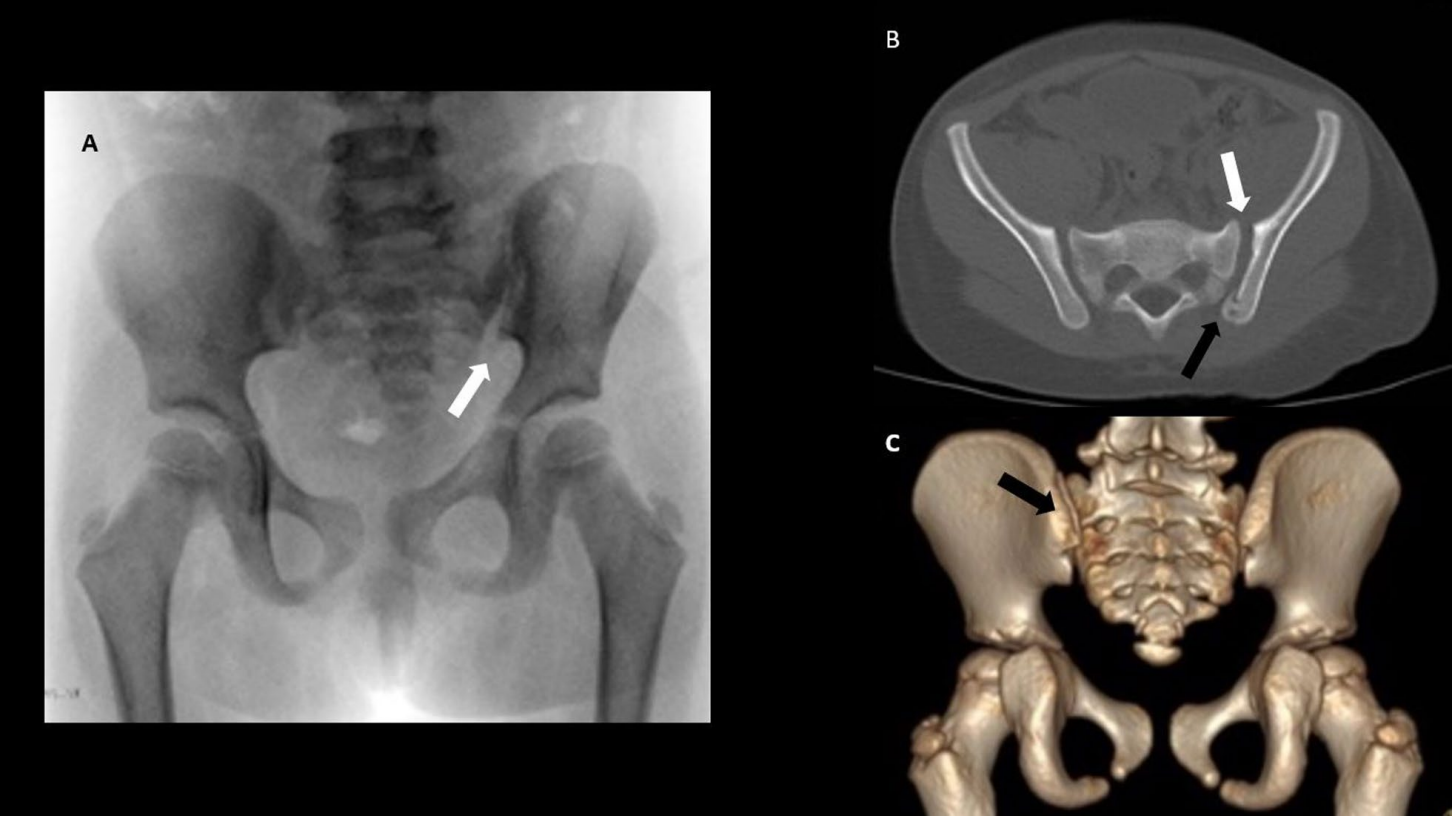
A type 1, Risser growth plate injury (RGPI). Notice the minimally displaced SI Joint (*arrow*) On the X rays the left hemipelvis appears intra-rotated respect to the right side. The CT scan shows a fracture line involving the posterior iliac spines. The three-dimensional (3D) image shows a fracture line that runs through the posterior iliac crest

Type 1: minimally displaced injuries without major sacroiliac (SI) joint involvement. This injury resembles a Salter–Harris type III physeal fracture and is biomechanically associated with internal rotation forces. The growth plate stays connected with the iliac crest, and posterior pelvic stability is preserved. (Fig. 2).

**Fig. 3:**
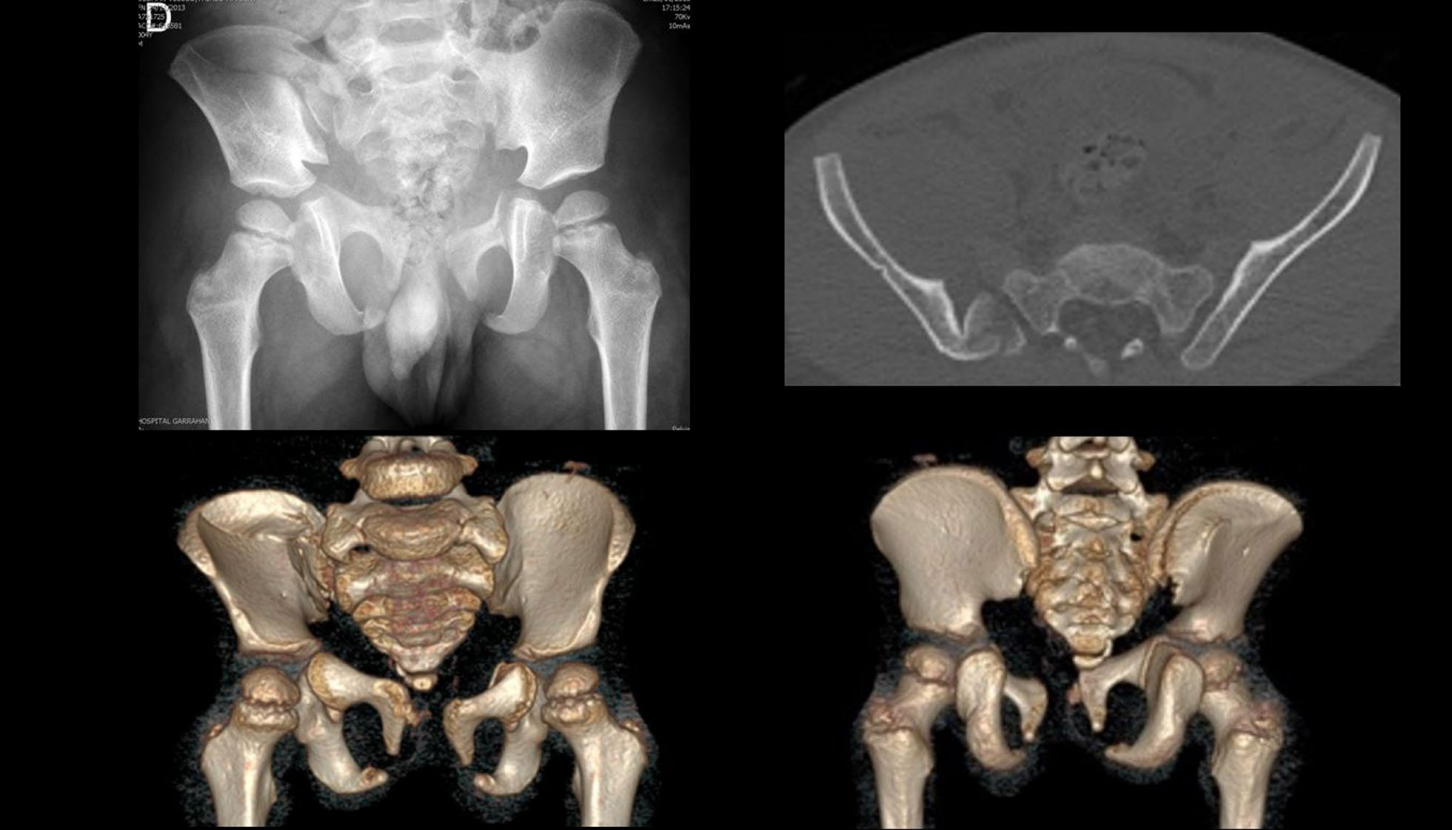
A type 2 Risser growth plate injury (RGPI). Notice the adduction and external rotation of the right hemipelvis demonstrated by the pubic symphysis deformity. The fracture line cuts the Risser growth plate through the iliac crest, but when seen in postero anterior view it is evident that there is an extensive separation of the iliac wing from the iliac crest if compared with the contra lateral side

Type 2: iliac wing fractures where an RGP segment is separated from the ilium with concomitant SI joint disruption (Salter–Harris type 2 equivalent), characterised by a positive ‘OK sign’ (Fig. 3).

**Fig. 4:**
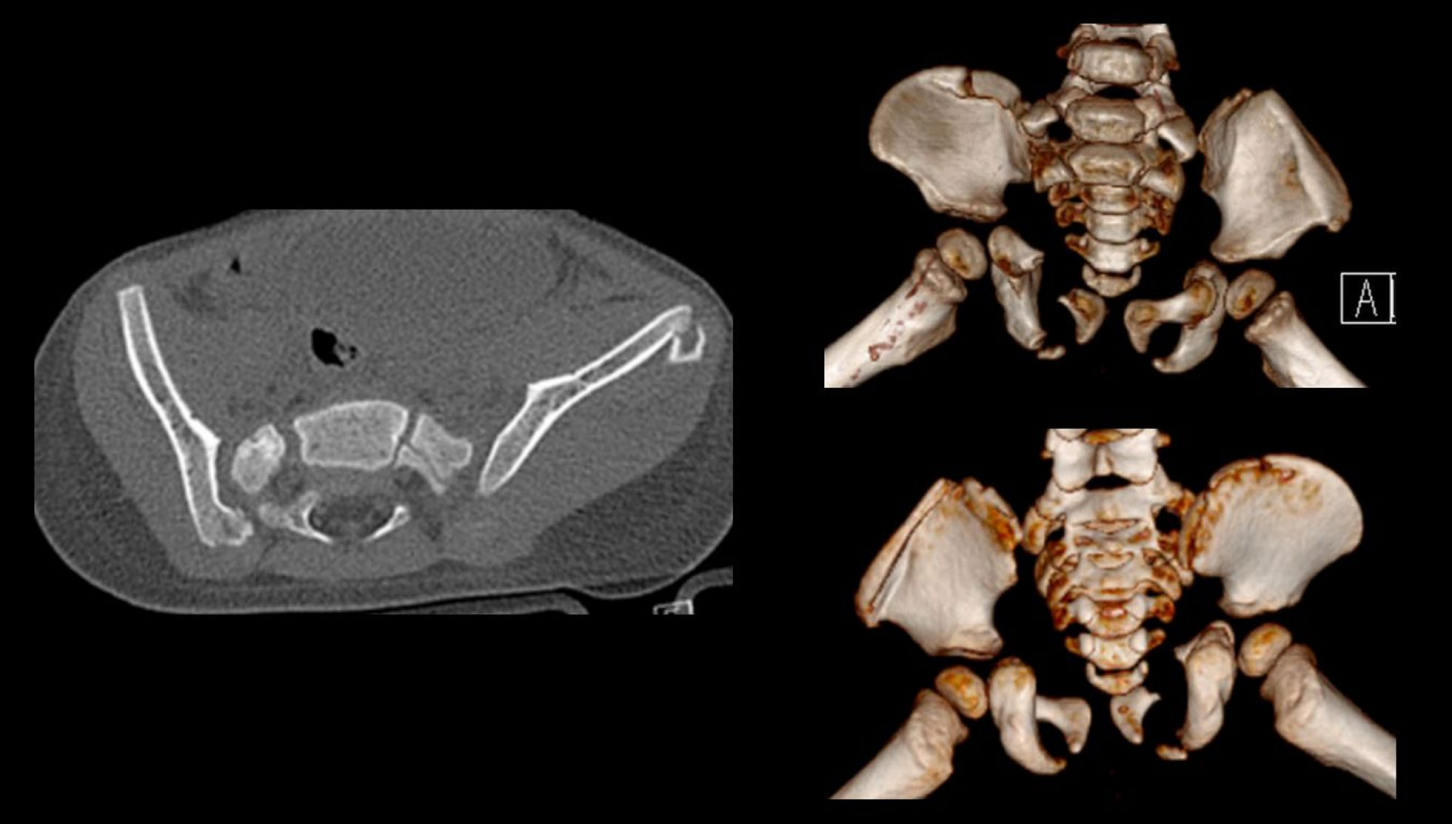
A type 3 Risser growth plate injury (RGPI), defined by contralateral or bilateral disruption. Shows a bilateral disruption sacro iliac joint disruption. On the right side an extended separation between and Risser growth plate and iliac crest is observed. On the left besides the SI joint involvement the iliac crest presents an extended bending that crosses the RGP at least in one point

Type 3: characterized by bilateral growth plate disruption, that can combine internal rotation in one side and vertical shear on the other side. This series also identified a novel variant that occurred in two children under 3 years of age who had bilateral SI joint dislocation (AO/OTA 61-C1.2) and, on one side, presented a greenstick fracture of the iliac wing that extended along the iliac crest from posterior to anterior and interrupted the RGP at a single point. Sacral ala compression fractures occurred in 28.6% (2/7) of bilateral cases owing to lateral compression mechanisms (Fig. 4).

### Treatment

The average time from injury to fixation was 10.1 days (range: 1–33 days; three cases were delayed more than 21 days owing to socio-economic factors). The surgical approach follows established principles of fracture management, using posterior fixation with 3.5- to 6.5-mm screws (sized to fit the sacral corridors). Reduced RGP nuclei were stabilised with K-wires. Anterior stabilisation was performed with plates, K-wires or external fixation, depending on the fracture configuration.

K-wires crossing the Risser apophysis were systematically removed between 60 and 90 days to avoid iatrogenic growth arrest.

### Follow-up

Only 13 patients (32.5%) had more than 2 years of follow-up. Eleven developed deformities ranging from minor limb length or volume discrepancies to severe growth arrest, caused by undergrowth of the hemipelvis rather than an alteration in lower limb growth. Imperfect percutaneous reductions – particularly in early cases where RGPI were unrecognised – and bilateral injuries contributed to asymmetries. Pelvic asymmetry was observed in four cases at 2, 5, 9 and 16 years post-trauma, with hemipelvic discrepancies ranging from 5 mm to 4 cm. Two patients exhibited sacral ala growth anomalies, resulting in secondary pelvic deformity. No disturbances in acetabular growth were noted (Table 3).

**Table 3:** Clinical characteristics, treatment and final deformity in patients with ≥2 years of follow-up (SI: Sacroiliac)

	Injury pattern (AO/OTA)	RGPI type	Treatment (fixation technique)		Final deformity (hemipelvis/sacrum/volume)
1	61-C3.1	Type 3 (bilateral)	Open	External fixation + bilateral SI screws	Minor hemipelvic volume discrepancy
2	61-C1.1	Type 2	Closed	Percutaneous SI screw	Hemipelvic discrepancy (4.0 cm)
3	61-C1.2	Type 2	Open	SI screw + K-wires for RGPI	None
4	61-C1.2	Type 2	Open	2 SI screws + K-wires for RGPI	Sacral ala growth anomaly (secondary deformity)
5	61-C3.1	Type 2	Open	SI screw + plate + Ramus screw	Minor hemipelvic volume discrepancy
6	61-C3.1	Type 3 (bilateral)	Open	External fixation + bilateral SI screws	Minor hemipelvic volume discrepancy
7	61-C1.2	Type 2	Open	Screws and plates + K-wires	None
8	61-C3.2	Type 3 (bilateral)	Open	3 SI screws + 3 K-wires for RGPI	Sacral ala growth anomaly (secondary deformity)
9	61-B2.2	Type 2	Open	SI screw + anterior plate	Minor hemipelvic volume discrepancy
10	61-C2.1	Type 2	Open	Bilateral SI screws + Ramus screws	Hemipelvic discrepancy (0.5 cm)
11	61-C3.1	Type 2	Open	SI screw + K-wires for RGPI	Minor hemipelvic volume discrepancy
12	61-B3.3	Type 2	Open	SI screw + anterior screw	Hemipelvic discrepancy (1.2 cm)
13	61-B2.1	Type 2	Conservative	Skeletal traction	Hemipelvic discrepancy (1.8 cm)

## Discussion

Unstable pelvic fractures in the immature skeleton represent a distinct clinical entity from adult pelvic trauma, principally owing to the vulnerability of the Risser growth plate (RGP) [6, 7, 16, 17].

It is crucial to emphasise that the paediatric pelvis differs from the adult pelvis solely because of the presence of growth plates (such as the Risser plate and triradiate cartilage). Biomechanically and anatomically, the pelvic ring behaves in the same way. However, these growth centres are specific points of vulnerability that determine unique fracture patterns (similar to Salter-Harris injuries) and require specialised surgical attention to avoid permanent growth disturbances.

This critical structure (RGPI) accounts for about 30% of pelvic growth and is injured in 65% of paediatric unstable pelvic fractures in our series (26/40 patients in this cohort). The incidence rises to 90.4% (19/21 cases) in AO/OTA type C fractures, emphasising that RGP injury (RGPI) constitutes an intrinsic component of high-energy paediatric pelvic trauma rather than a coincidental finding.

Our data demonstrate that injury patterns correlate strongly with specific mechanisms: internal rotation forces typically produced minimally displaced type 1 injuries without sacroiliac joint disruption; combined shear-compression mechanisms lead to type 2 iliac wing avulsions with pathognomonic ‘OK sign or obturator ring sign’ presentation; and high-energy lateral compression generated type 3 bilateral injuries, including a novel pattern in children under 3 years, featuring bilateral sacroiliac dislocations with contralateral iliac greenstick fractures.

Our data emphasises a key principle in paediatric orthopaedics: epiphyseal detachments must be anatomically realigned. Standard percutaneous fixation, although effective in providing adult-like mechanical stability, often failed to achieve anatomical reduction of the RGP in our paediatric cohort. As a result, poor reduction led to a high rate (84.6%) of progressive functional deformities, with LLD (due to hemipelvic discrepancies) ranging from 5 to 40 mm, rather than resulting in joint instability [18].

This progression arises from three interconnected factors: firstly, the intrinsic severity of RGPI as a Salter–Harris equivalent that disrupts physeal function [19]; secondly, imperfect reduction, especially with percutaneous techniques that failed to correct RGP displacement (evidenced by hemipelvic discrepancies ranging from 5 mm to 4 cm); and thirdly, the combined effect of bilateral injuries leading to symmetric growth arrest. These observations, as described by Oransky, confirm that RGPI is the main cause of post-traumatic pelvic deformity [6]. Younger children (<7 years) may face increased risks owing to their higher growth potential, as seen with physeal injuries in other parts of the skeleton [20].

**Fig. 5:**
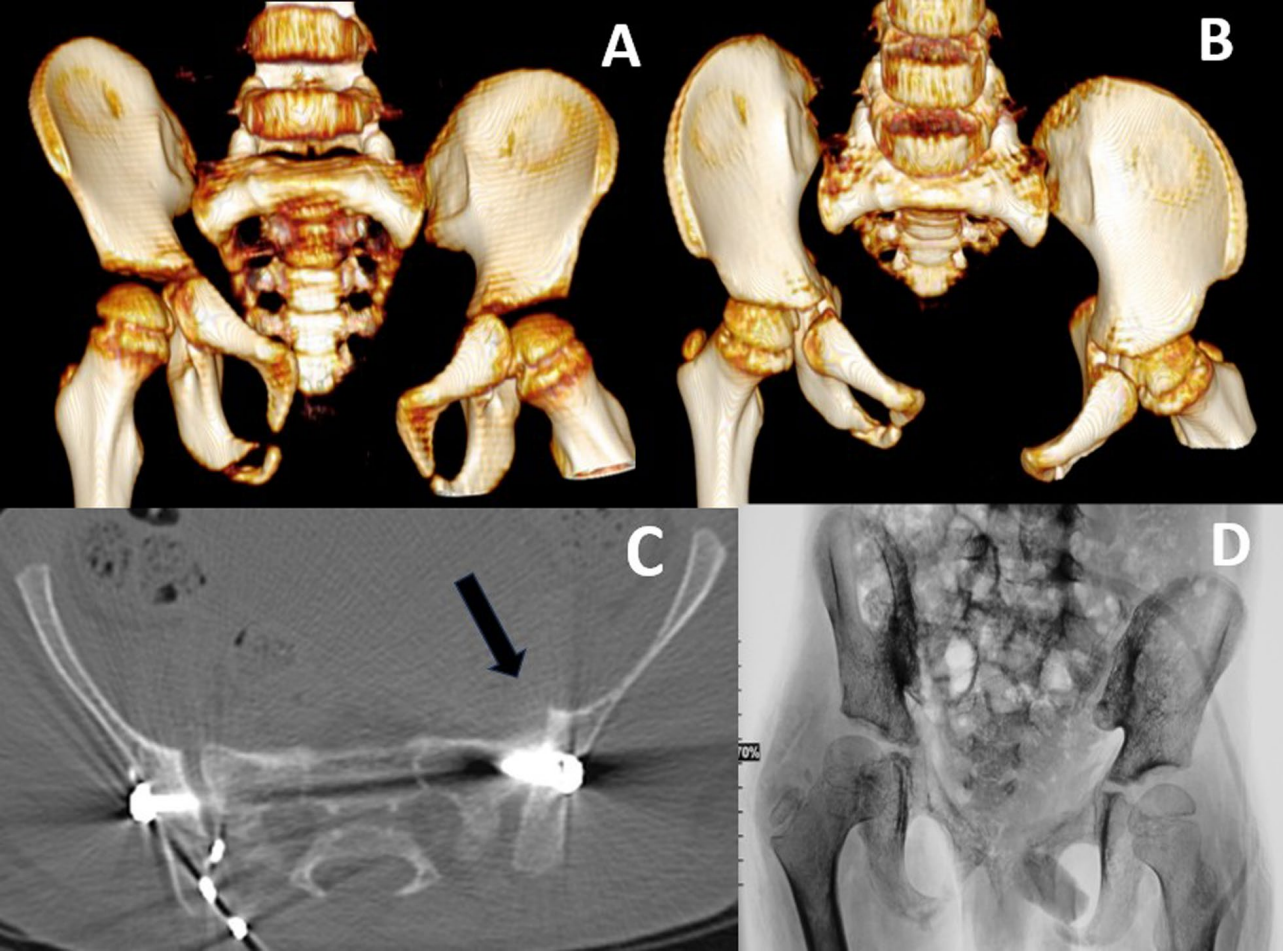
Rolled-over patient with bilateral sacroiliac joint dislocation, more displaced on the right side. It was associated anteriorly with pubic symphysis disruption. **A** The 3D AP view. **B** The 3D inlet view. The patient underwent surgical treatment on the right side using an anatomical approach for adequate repositioning of the Risser growth plate. The left side was fixed percutaneously after a closed reduction, without addressing the Risser growth plate. **C** The imperfect reduction of the sacroiliac joint (*arrow*). **D** The anteroposterior radiograph at the 5-year follow-up, demonstrating a severe growth disturbance of the iliac wing

Compression fractures of the sacral ala, identified in 20% (8/40) of our cohort, warrant particular concern. As established by Oransky et al., these represent irreversible crushing injuries to growth nuclei caused by trabecular impaction [6]. Their irreducibility explains the persistent deformity observed despite attempts at fixation. This phenomenon supports Schwarz et al.’s report of 47% poor outcomes associated with pelvic asymmetry in non-anatomically reduced fractures [21]. Our experience further demonstrates that percutaneous fixation proves fundamentally inadequate for managing RGPI, as it cannot reduce or stabilise the growth plate, consistently leading to severe growth disturbances, such as marked hemipelvic hypoplasia (Fig. 5) [22].

Delayed intervention (>21 days) in three socio-economically disadvantaged patients further reduced the quality of the reduction.

This study has several strengths. It represents the largest reported cohort of RGPIs to date. A noteworthy and distinctive discovery of this multicentre study is the identification of RGPI in type B fractures. To our knowledge, this is the sole study in the current orthopaedic literature to document Risser growth plate injuries in type B (partially stable) lesions. This indicates that the RGP can be avulsed or displaced by rotational forces (type B), not only by complete vertical or translational disruptions (type C). This discovery emphasises the importance of maintaining a high level of suspicion and conducting a systematic CT scan of the entire pelvis, including the iliac crest, in all paediatric pelvic ring injuries, regardless of the apparent stability of the primary fracture pattern.

However, we acknowledge several limitations in the current study. Primarily, the retrospective multicentre design and the extended inclusion period spanning 35 years introduce inherent methodological heterogeneity, particularly regarding the evolution of treatment protocols and imaging techniques over time. Second, of the 40 children included, only 13 (32.5%) were available for long-term (>2 years) clinical and radiographic follow-up, mainly owing to socio-economic factors in some centres. While this relatively small sub-cohort restricts the ability to perform extensive statistical correlations between all injury characteristics and treatment modalities, the findings remain highly significant. The striking correlation between unreduced Risser growth plate injuries (RGPIs) and the subsequent development of severe structural deformities – observed in 11 of 13 patients – provides critical, actionable clinical insights. Future prospective studies with larger paediatric cohorts and standardised follow-up protocols are warranted to further validate our proposed classification and refine surgical algorithms.

Nevertheless, this constitutes the largest RGPI cohort reported to date, providing critical insights into injury patterns and treatment failures. We argue that open reduction with direct visualisation and stabilisation of the RGP is essential for displaced fractures. Achieving anatomical growth plate alignment must be equally prioritised alongside osseous reduction to prevent the documented sequelae of pelvic asymmetry, leg-length discrepancy and functional impairment. Future prospective multicentre studies should standardise operative protocols, correlate RGPI subtypes with growth arrest patterns and establish socio-economic support systems to enhance long-term monitoring.

## Conclusions

Risser growth plate injury (RGPI) is an essential, inherent part of displaced, unstable paediatric pelvic fractures, occurring in 65% of patients and increasing to 90.4% in AO/OTA type C injuries.

The high occurrence of RGPI, classified into three distinct patterns linked to specific injury mechanisms, highlights its vital role as a primary factor in post-traumatic growth disturbance, especially in cases of bilateral injuries. Sacral ala compression fractures, seen in 20% of cases, indicate irreversible physeal damage. Given the high rate of deformity (84.6%) observed in our follow-up cohort, we conclude that percutaneous fixation alone is inadequate for treating this condition. Anatomic open reduction and direct stabilisation of both the bone and the Risser growth plate are essential to prevent long-term sequelae.

## Data Availability

The data supporting the findings of this study are available from the corresponding author upon reasonable request.
